# Effect of liraglutide on thigh muscle fat and muscle composition in adults with overweight or obesity: Results from a randomized clinical trial

**DOI:** 10.1002/jcsm.13445

**Published:** 2024-04-01

**Authors:** Ambarish Pandey, Kershaw V. Patel, Matthew W. Segar, Colby Ayers, Jennifer Linge, Olof D. Leinhard, Stefan D. Anker, Javed Butler, Subodh Verma, Parag H. Joshi, Ian J. Neeland

**Affiliations:** ^1^ University of Texas Southwestern Medical Center Dallas TX USA; ^2^ Department of Cardiology Houston Methodist DeBakey Heart & Vascular Center Houston TX USA; ^3^ Department of Cardiology Texas Heart Institute Houston TX USA; ^4^ AMRA Medical and Linköping University Linköping Sweden; ^5^ Department of Health, Medicine and Caring Sciences, Division of Diagnostics and Specialist Medicine Linköping University Linköping Sweden; ^6^ Department of Cardiology (CVK), Berlin Institute of Health Center for Regenerative Therapies (BCRT), and German Centre for Cardiovascular Research (DZHK) Partner Site Berlin Charité Universitätsmedizin Berlin Germany; ^7^ Baylor Heart and Vascular Institute Baylor University Medical Center Dallas TX USA; ^8^ Department of Medicine University of Mississippi School of Medicine Jackson MS USA; ^9^ St. Michael's Hospital University of Toronto Toronto ON Canada; ^10^ Harrington Heart and Vascular Institute University Hospitals Cleveland Medical Center and Case Western Reserve University School of Medicine Cleveland OH USA

**Keywords:** Body composition, Liraglutide, Muscle fat, Muscle volume, Obesity, Overweight

## Abstract

**Background:**

Excess muscle fat is observed in obesity and associated with greater burden of cardiovascular risk factors and higher risk of mortality. Liraglutide reduces total body weight and visceral fat but its effect on muscle fat and adverse muscle composition is unknown.

**Methods:**

This is a pre‐specified secondary analysis of a randomized, double‐blind, placebo‐controlled trial that examined the effects of liraglutide plus a lifestyle intervention on visceral adipose tissue and ectopic fat among adults without diabetes with body mass index ≥30 kg/m^2^ or ≥27 kg/m^2^ and metabolic syndrome. Participants were randomly assigned to a once‐daily subcutaneous injection of liraglutide (target dose 3.0 mg) or matching placebo for 40 weeks. Body fat distribution and muscle composition was assessed by magnetic resonance imaging at baseline and 40‐week follow‐up. Muscle composition was described by the combination of thigh muscle fat and muscle volume. Treatment difference (95% confidence intervals [CI]) was calculated by least‐square means adjusted for baseline thigh muscle fat. The association between changes in thigh muscle fat and changes in body weight were assessed using Spearman correlation coefficients. The effect of liraglutide versus placebo on adverse muscle composition, denoted by high thigh muscle fat and low thigh muscle volume, was explored.

**Results:**

Among the 128 participants with follow‐up imaging (92.2% women, 36.7% Black), median muscle fat at baseline was 7.8%. The mean percent change in thigh muscle fat over median follow‐up of 36 weeks was −2.87% among participants randomized to liraglutide (*n* = 73) and 0.05% in the placebo group (absolute change: −0.23% vs. 0.01%). The estimated treatment difference adjusted for baseline thigh muscle fat was −0.24% (95% CI, −0.41 to −0.06, *P*‐value 0.009). Longitudinal change in thigh muscle fat was significantly associated with change in body weight in the placebo group but not the liraglutide group. The proportion of participants with adverse muscle composition decreased from 11.0% to 8.2% over follow‐up with liraglutide, but there was no change with placebo.

**Conclusions:**

In a cohort of predominantly women with overweight or obesity in the absence of diabetes, once‐daily subcutaneous liraglutide was associated with a reduction in thigh muscle fat and adverse muscle composition compared with placebo. The contribution of muscle fat improvement to the cardiometabolic benefits of liraglutide requires further study.

## Introduction

Obesity affects 42.4% of adults in the United States and contributes to the development of atherosclerotic cardiovascular disease, heart failure (HF), and atrial fibrillation.^1^, ^2^ Body mass index (BMI) is commonly used to define obesity but does not differentiate between muscle and fat or distinguish between volume of distribution of these metabolically active tissues.^3^ Lean mass, visceral adipose tissue, subcutaneous adipose tissue, and ectopic fat, including muscle fat, differentially impact cardiometabolic risk through their heterogeneous effects.^2^, ^3^, ^4^ Muscle fat is an important marker of risk given its associations with impaired functional capacity,^5^, ^6^ cardiometabolic risk factors,^7^, ^8^ and development of cardiovascular disease.^9^, ^10^, ^11^ Adverse muscle composition, denoted by high muscle fat combined with low muscle volume, has been linked to poor function, comorbidity, and risk of hospitalization and is a strong and independent predictor of all‐cause mortality.^12^, ^13^, ^14^ However, whether muscle fat and adverse muscle composition are modifiable targets for therapeutic interventions is not well established.

The first‐line therapy for obesity management is lifestyle modification consisting of dietary changes and physical activity counselling.^15^ Major limitations of lifestyle modification for obesity management includes the modest amount of weight loss achieved, high rates of weight regain over follow‐up, and null effects for preventing cardiovascular disease events.^16^ Medications for weight loss serve as an adjunctive therapy with select agents demonstrating large and sustainable reductions in weight.^15^ Liraglutide is a glucagon‐like peptide 1 receptor agonist (GLP1RA) that reduces appetite, decelerates gastric emptying, and leads to approximately 5 kg weight loss with sustained effects up to 3 years.^17^, ^18^, ^19^ Additionally, liraglutide demonstrated beneficial effects on cardiovascular disease among patients with type 2 diabetes at elevated cardiovascular disease risk in a large, randomized clinical trial.^20^ Liraglutide has several favourable cardiometabolic effects, including reducing body weight and visceral adiposity, but its effects on muscle fat and adverse muscle composition are not well established.^18^, ^21^ Previous studies of liraglutide focused predominantly on patients with type 2 diabetes and demonstrated favourable effects on weight loss and body composition improvement.^22^, ^23^, ^24^ However, its effects on body composition, particularly regional adiposity depots in a population without type 2 diabetes, remain largely undefined.

In the present study, we examined the effects of liraglutide on muscle fat and adverse muscle composition as identified by magnetic resonance imaging (MRI) in a secondary analysis of a randomized, double‐blind, placebo‐controlled trial in adults free from diabetes living with overweight or obesity and high cardiovascular risk. We hypothesized that liraglutide would reduce muscle fat and adverse muscle composition in this population.

## Methods

### Study overview: Design, population, and procedures

The present study is a pre‐specified secondary analysis of a randomized, double‐blind, placebo‐controlled clinical trial that evaluated the effects of liraglutide on visceral and ectopic fat among adults without diabetes who had overweight or obesity and high cardiovascular risk (ClinicalTrials.gov: NCT03038620). Details of the clinical trial have been published previously.^21^ In brief, the trial enrolled adults ≥35 years of age with overweight or obesity (BMI ≥ 27 kg/m^2^ with metabolic syndrome or BMI ≥ 30 kg/m^2^) who were free of type 1 or type 2 diabetes. Metabolic syndrome was defined using the National Cholesterol Education Program Adult Treatment Panel III criteria and was based on the presence of at least three of the following: (1) abdominal obesity (waist circumference >88 cm in women or >102 cm in men); (2) hypertriglyceridaemia (fasting triglyceride level ≥150 mg/dL or treatment for this); (3) low high‐density lipoprotein cholesterol (<50 mg/dL in women or <40 mg/dL in men); (4) hyperglycaemia (fasting glucose ≥100 mg/dL); (5) elevated blood pressure (≥130/85 mm Hg or treatment for hypertension).^25^ Diabetes history was based on self‐reported medical history and haemoglobin A1c ≥ 6.5%. Participants must have been able to complete body fat assessment with MRI (no contraindications such as presence of a cardiac implantable electronic device or metallic fragments). Exclusion criteria included the following: current or planned use of obesity therapy (including GLP1RA), contraindication for GLP1RA use (such as hypersensitivity to liraglutide, personal or family history of medullary thyroid carcinoma or multiple endocrine neoplasia syndrome type 2), high risk for serious adverse effect from GLP1RA, pregnancy, or breast feeding. The present study included participants who underwent randomization and had interpretable MRI imaging for body composition analysis at baseline and follow‐up (Figure S1). Individuals were recruited from the University of Texas Southwestern Medical Center from 2017 to 2020. The institutional review board at the University of Texas Southwestern Medical Center approved the clinical study. All participants provided written informed consent.

After screening, participants underwent a 2‐week run‐in phase of diet and physical activity counselling. Counselling on diet involved prescription of a hypocaloric diet. Baseline total energy expenditure was estimated and a 500‐kcal deficient diet was prescribed that included the following energy sources: 30% fat, 20% protein, 50% carbohydrates. Physical activity counselling included a target of ≥150 min per week of moderate‐intensity activity. Physical activity was assessed at baseline and participants were counselled to increase physical activity to recommended levels based on the discretion of the participant. Adherence to dietary counselling was assessed with 3‐day food diaries during the run‐in phase and periodically after randomization. Participants were excluded if they were unable to adhere to the diet and physical activity recommendations during the run‐in phase. After the run‐in phase, participants were randomly assigned in a 1:1 ratio to receive a subcutaneous injection of liraglutide or a matching placebo. Randomization was performed using a computerized randomization code that was created by a statistician not involved in the conduct of the study at the University of Texas Southwestern Medical Center. Liraglutide or matching placebo was self‐administered subcutaneously once daily using a pen injector with an adjustable dose indicator. Participants were started on 0.6 mg of liraglutide or matching placebo once daily with up‐titration by 0.6 mg weekly as tolerated to a maximum dose of 3.0 mg. The treatment protocol duration was 40 weeks. Participants, study personnel, and adjudicators of outcomes were unaware of the treatment assignment until study completion. Among participants with analysable dietary recall data, baseline calorie and macronutrient consumption were similar across treatment groups. Participants appeared to adhere to the diet recommendations as evidenced by a decline in total calorie and macronutrient consumption throughout the trial period with no significant interactions by treatment assignment.

### Study covariates

Participants completed standardized interviews and questionnaires.^21^ Age, sex, race/ethnicity, and medical history were self‐reported. Height and weight were measured while participants were shoeless and wearing loose fitting clothing using standard scales. BMI was calculated by dividing weight in kg by height in m^2^. Waist circumference was measured using a tape measure at 1 cm above the iliac crest. An automatic blood pressure cuff (Omron 5 series) was used to measure blood pressure and heart rate (Omron Healthcare, Lake Forest, IL, USA). Hypertension was defined as blood pressure ≥140/90 mmHg. Fasting blood samples were obtained and standard assays at the University of Texas Southwestern Medical Center were used to measure levels of triglyceride, high‐density lipoprotein cholesterol, and glucose. Hyperlipidaemia was defined as high‐density lipoprotein cholesterol <40 mg/dL or triglyceride >200 mg/dL. These serum biomarkers were measured at baseline and follow‐up at 40 weeks.

### Primary outcome and body composition analysis

The primary outcome of the present study was percent change in thigh muscle fat over 40 weeks assessed by MRI. All participants were imaged from neck to knees using a standardized, rapid (6–10 min) two‐point Dixon protocol in a 3‐Tesla MRI scanner (Philips Achieva, Philips Healthcare, Amsterdam, Netherlands; or General Electric 750 wide bore, GE Healthcare, Chicago, IL).^21^ Image analysis was done using AMRA Researcher (AMRA Medical AB, Linköping, Sweden).^26^, ^27^, ^28^, ^29^ Briefly, the analysis includes (1) calibration of images using fat‐referenced MRI based on the fat within the image itself, using the adipose tissue of each scan as an internal reference,^26^, ^29^ (2) generation of automatic segmentations using registration of atlases with ground truth labels for fat‐ and muscle compartments, (3) quality control of the automatic segmentation by two trained and independent operators with anatomical knowledge and the possibility to assess potential image quality issues as well as adjust segmentations if needed, and (4) quantification of fat‐ and muscle volumes, as well as muscle fat. The same scanner was used for both baseline and 40‐week follow‐up. Both scanners used a similar neck‐to‐knee protocol using multiple overlapping two‐point Dixon spoiled GRE sequences (mDixon and LAVA‐Flex, respectively) and a dedicated proton density fat fraction sequence for liver fat assessment. The main MRI protocol parameters were as follows: flip angle 10°, repetition time set to shortest, echo times set automatically, and phase‐encoding direction anterior–posterior. The acquired voxel sizes were 2.1 × 2.0 × 5.2 mm^3^ (abdomen) and 2.1 × 1.7 × 3 mm^3^ (thighs) on the Philips scanner, and 2.5 × 2.1 × 5 mm^3^ (abdomen) and 1.9 × 1.5 × 4 mm^3^ (thighs) on the General Electric scanner. Previous validation has shown high reproducibility and repeatability across both field strengths and manufacturers.^28^

*Muscle volume* was measured as ‘fat‐free muscle volume’, defined as the total volume of image voxels with fat fraction below 50% (sometimes referred to as ‘viable muscle tissue’) within the segmented regions.^26^ Muscle volume was assessed in the anterior (including qaudriceps femoris, sartorius, and tensor fascia latae) and posterior (gluteus muscles, iliacus, adductor muscles, and hamstring muscles) compartments of the thighs.

*Muscle fat* was measured as ‘muscle fat infiltration’, defined as the T2*‐corrected proton density fat fraction of the fat‐free muscle volume.^28^, ^29^ Muscle fat was assessed in the anterior thighs and the average from the bilateral thighs was used in this analysis. The anterior compartment of the thigh muscle was examined as this consists primarily of the quadriceps muscle and has lower variability in muscle fat infiltration at baseline as well as over follow‐up compared with the posterior compartment that contains several muscles.^29^ Other body composition parameters that were assessed include total lean tissue volume, visceral adipose tissue volume, and abdominal‐ and pelvic/thigh subcutaneous adipose tissue volume.

*Adverse muscle composition* was an exploratory outcome and was defined as high muscle fat (mean anterior thigh muscle fat infiltration >75th percentile for each sex: men >7.69%, women >8.82%) plus low muscle volume (<25th percentile, muscle volume *z*‐score <−0.68).^12^, ^14^ The muscle volume *z*‐score is a sex‐, height‐, and weight invariant variable describing how much each participant deviates (in standard deviations) from (at least *N* = 150) sex‐ and BMI‐matched individuals.^13^ For matching, data from the UK Biobank imaging study was used.^14^ Participants with low muscle fat and high muscle volume *z*‐score were classified as having ‘normal’ muscle composition. Additionally, participants could be classified as having either only high muscle fat or only low muscle volume *z*‐score.

### Statistical analysis

Baseline and follow‐up characteristics of the study population were compared across data‐derived tertiles of muscle fat at baseline using chi‐square test for categorical variables and Kruskal–Wallis for continuous variables. The effect of liraglutide versus placebo on percent change in muscle fat, muscle volume, and muscle volume *z*‐score was assessed using least square means adjusted for baseline levels, respectively. The median (95% CI) percent change in muscle fat and muscle volume over 40 weeks was reported for participants by treatment group. Subgroup analyses were performed examining differences in percent change of muscle fat using a Kruskal–Wallis test among participants stratified by age (below and above median), race/ethnicity (White, Black, and Other), and BMI categories (overweight, class 1 obesity, class 2 or 3 obesity). Multiplicative interaction testing was performed for subgroups. The absolute change in muscle fat over 40 weeks across treatment groups was also reported. The association of liraglutide treatment with change in muscle fat and muscle volume was assessed with linear regression models. Separate models were created for each outcome (follow‐up measure of muscle fat or muscle volume adjusted for the baseline value) with the inclusion of the following covariates: *Model 1* : age, sex, race, baseline BMI, treatment group, and the baseline measure of the outcome. To evaluate whether the effect of liraglutide on change in muscle fat was independent of changes in other body composition parameters, the following models were also constructed: *Model 2* : Model 1 covariates plus absolute change in total lean tissue volume; *Model 3* : Model 2 covariates plus absolute change in visceral fat. Participants were also stratified by tertiles of percent change in muscle fat, and baseline characteristics were compared as described above. Independent predictors of follow‐up muscle fat were assessed using linear regression analysis with adjustment for age, sex, race, BMI, systolic blood pressure, fasting plasma glucose, high‐density lipoprotein cholesterol, and baseline muscle fat.

The associations of longitudinal change in body composition parameters with change in muscle fat on follow up were assessed using linear mixed effect models with adjustments for age, sex, race, treatment group, pre‐/post‐period, and patient ID as a random intercept. To evaluate muscle fat and muscle volume *z*‐score changes in relation to weight changes across treatment groups, the change in muscle fat/muscle volume *z*‐score was plotted with the change in body weight for the liraglutide and placebo groups separately. The Spearman correlation coefficient was calculated to assess the association of change in body weight with change in muscle fat/volume z‐score across treatment groups.

In exploratory analysis, the proportion of participants with normal and adverse muscle composition, only high muscle fat, and only low muscle volume were described at baseline and 40‐week follow‐up in the liraglutide and placebo treatment groups. Missing data were imputed using random forest imputation (only total lean tissue had 1% missingness).^30^ A two‐sided *P*‐value <0.05 was considered statistically significant. SAS version 9.4 (SAS Corporation, Cary, NC) or R 4.0.3 (R Foundation, Vienna, Austria) was used for all statistical analyses.

## Results

This secondary analysis included 128 participants randomized to liraglutide (*n* = 73) or placebo (*n* = 55) with interpretable baseline and follow‐up body composition imaging with MRI (Figure S1). Baseline and follow‐up levels of muscle fat and other characteristics of participants stratified by treatment group are shown in Table S1. In the overall study population, 92.2% were women and 36.7% were Black. Muscle fat ranged from 4.5% to 14.1% with a median of 7.8% at baseline. The baseline characteristics of participants across tertiles of muscle fat assessed at baseline are shown in Table 1. Participants with higher muscle fat had higher levels of overall obesity as well as greater amounts of visceral adipose tissue and subcutaneous adipose tissue. There were no significant differences in age, sex, race/ethnicity, history of hypertension or blood pressure levels, or total lean tissue across tertiles of muscle fat. Follow‐up measures of adiposity, specifically BMI, visceral adipose tissue, subcutaneous adipose tissue, and muscle fat remained higher among participants with greater muscle fat at baseline, but there were no significant differences in glycaemic or lipid levels (Table S2). Across tertiles of change in muscle fat, there were no major differences in baseline BMI, visceral adipose tissue, blood pressure, or comorbid conditions (Table S3).

**Table 1:** Baseline characteristics of participants stratified by tertiles of baseline muscle fat.

	Tertile 1 (*n* = 43)	Tertile 2 (*n* = 43)	Tertile 3 (*n* = 42)	*P*‐value
Mean thigh muscle fat infiltration, %	6.2 (5.7, 6.6)	7.8 (7.5, 8.1)	10.0 (9.2, 10.8)	<0.001
Age, years	48 (41, 55)	49 (43, 56)	52 (44, 57)	0.40
Female	39 (90.7)	37 (86.0)	42 (100)	0.05
Race (%)				0.20
White	20 (46.5)	30 (69.8)	28 (66.7)	
Black	22 (51.2)	12 (27.9)	13 (31.0)	
Systolic blood pressure, mmHg	126 (117, 142)	126 (114, 134)	131 (120, 139)	0.44
Diastolic blood pressure, mmHg	79 (73, 84)	79 (73, 87)	80 (76, 86)	0.73
Weight, kg	92.4 (82.2, 105.3)	98.3 (86.7, 115.8)	109.0 (98.3, 124.6)	0.001
BMI, kg/m^2^	35.2 (31.7, 37.0)	37.3 (33.4, 40.4)	40.1 (36.2, 44.7)	<0.001
Height, m	1.6 (1.6, 1.7)	1.6 (1.6, 1.7)	1.7 (1.6, 1.7)	0.94
Medical history				
Hyperlipidaemia	14 (32.6)	23 (53.5)	25 (59.5)	0.03
Hypertension	13 (30.2)	8 (18.6)	12 (28.6)	0.41
Laboratory values				
Fasting plasma glucose, mg/dL	100 (92, 105)	98 (93, 107)	97 (91, 106)	0.91
HDL‐C, mg/dL	59 (54, 65)	54 (47, 58)	56 (47, 64)	0.21
Triglycerides, mg/dL	110 (83, 149)	106 (78, 148)	98 (76, 124)	0.41
Body composition parameter				
Visceral adipose tissue, L	3.8 (2.7, 4.7)	4.3 (3.4, 6.1)	4.6 (3.7, 5.6)	0.01
Abdominal subcutaneous adipose tissue, L	14.2 (12.0, 15.8)	15.6 (11.4, 18.3)	18.0 (15.5, 20.5)	<0.001
Total thigh muscle, L	10.0 (9.3, 11.1)	9.4 (8.4, 10.9)	9.4 (8.6, 10.4)	0.18
Total lean tissue, L	21.0 (19.6, 23.7)	21.1 (18.7, 23.6)	21.4 (19.0, 23.3)	0.91
Thigh muscle volume *z*‐score	0.8 (0.2, 1.6)	‐0.1 (−0.7, 0.6)	−0.2 (−1.0, 0.6)	<0.001

Categorical variables are described as number (%) and compared using chi‐square test. Continuous variables are described as median (25–75%) and compared using Kruskal–Wallis test.

BMI, body mass index; HDL‐C, high‐density lipoprotein cholesterol.

**Figure 1:**
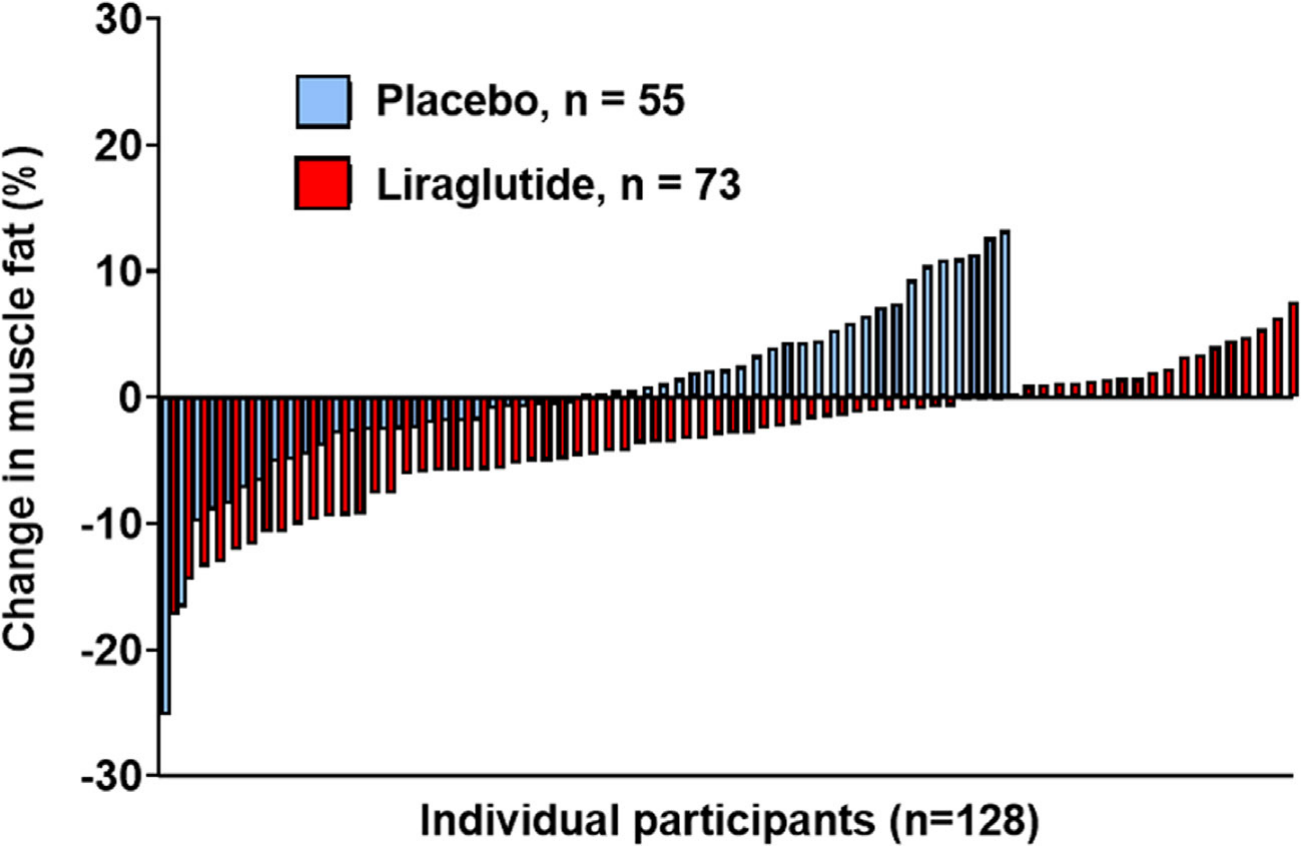
Participant‐level relative change in muscle fat across treatment groups. Individual participant percent change in muscle fat is shown for all participants randomized to liraglutide (red) and placebo (blue). Individual participants were ordered on the x‐axis from greatest relative decrease to increase in muscle fat.

The relative change in muscle fat for each participant in the liraglutide and placebo groups over a median follow‐up of 36 weeks is shown in Figure 1. In the liraglutide group, 51 of 73 participants (69.9%) had a reduction in muscle fat during follow‐up, whereas a reduction in muscle fat was observed in only 27 of 55 participants (49.1%) randomized to placebo. At least a 10% reduction in muscle fat was observed among 12.3% and 3.6% of participants in the liraglutide and placebo groups, respectively. Liraglutide reduced mean muscle fat from baseline to 40‐week follow‐up compared with placebo with least‐square means difference adjusted for baseline muscle fat of −0.24% (95% CI: −0.41, −0.06, *P*‐value = 0.009; absolute change: −0.23% [95% CI: −0.48, 0.05] vs. 0.01% [95% CI: −0.23, 0.36], *P*‐value = 0.001; percent change: −2.87% [95% CI: −5.78, 0.37] vs. 0.05% [−2.58, 4.35], *P*‐value = 0.001). The effect of liraglutide on muscle fat was consistent across subgroups stratified by age, race/ethnicity, and BMI categories (Table 2). In adjusted regression analysis accounting for demographics and baseline BMI, liraglutide treatment was associated with lower muscle fat on follow‐up (β estimate [95% CI] in Model 1: −0.20 [−0.37, −0.03], *P* = 0.02). This association was consistent after additional adjustment for change in total lean tissue volume (β estimate [95% CI] in Model 2: −0.25 [−0.42, −0.07], *P*‐value = 0.007) but not after further adjustment for change in visceral adipose tissue (β estimate [95% CI] in Model 3: −0.10 [−0.28, 0.08], *P*‐value = 0.30).

**Table 2:** Effect of liraglutide on percent change in muscle fat across subgroups.

	Placebo		Liraglutide		*P*‐value	*P*‐int
	Number	Change in muscle fat (%), median (IQR)	Number	Change in muscle fat (%), median (IQR)		
Age						0.29
≤Median	27	0.05 [−3.32, 3.41]	39	−3.55 [−5.81, 0.66]	0.06	
>Median	28	0.04 [−1.95, 5.48]	34	−2.08 [−5.78, 0.06]	0.004	
Race/ethnicity						0.08
White	35	−0.76 [−4.19, 2.35]	43	−2.87 [−5.97, −0.42]	0.07	
Black	19	2.00 [−0.86, 7.32]	28	−1.36 [−5.73, 1.19]	0.002	
Other	1	−0.44 [−0.44, −0.44]	2	−8.69 [−10.39, −6.99]	0.22	
BMI categories						0.46
Overweight	4	−3.30 [−5.20, −1.04]	6	−9.67 [−11.59, −5.49]	0.14	
Obese, class 1	33	0.86 [−1.90, 5.35]	46	−3.09 [−5.78, 0.74]	0.006	
Obese, class 2/3	18	0.08 [−2.58, 3.11]	21	−1.70 [−5.06, 1.15]	0.14	

Median (interquartile range) percent change in muscle fat was calculated for each treatment group. Kruskal–Wallis test was used to evaluate for differences in percent change in muscle fat across treatments in subgroups. Multiplicative interaction testing was performed for subgroups. For cells with *n* < 5, Fisher's exact test was used to make comparisons.

BMI, body mass index; IQR, interquartile range; MFI, muscle fat infiltration.

In adjusted analysis, higher baseline BMI and muscle fat were both significantly associated with higher muscle fat on follow‐up (Table S4). During follow‐up, liraglutide reduced muscle volume compared with placebo with least‐square means difference adjusted for baseline muscle volume of −0.29 L (95% CI, −0.42, −0.16, *P*‐value <0.001; percent change: −3.18% vs. −0.11%, respectively). Liraglutide treatment was associated with lower muscle volume on follow‐up after accounting for demographics and baseline BMI (β estimate [95% CI] in Model 1: −0.30 [−0.43, −0.17], *P* < 0.001). The change in muscle volume *z*‐score was similar across liraglutide and placebo treatment groups with least‐square means difference adjusted for baseline muscle volume *z*‐score of −0.08 (95% CI, −0.20 to 0.04, *P*‐value 0.18).

**Figure 2:**
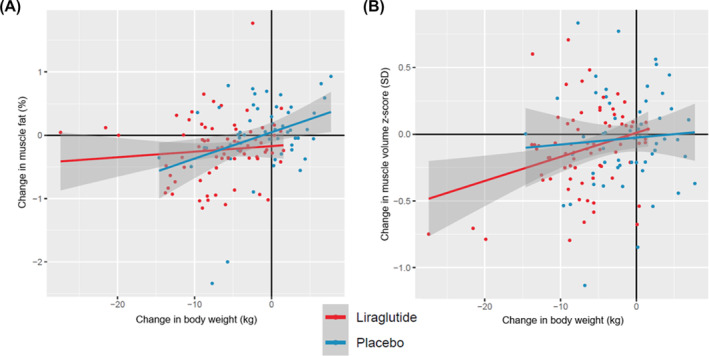
Association of change in body weight with change in muscle fat (panel A) or muscle volume *z*‐score (panel B) across treatment groups. The absolute difference in muscle fat and muscle volume *z*‐score from baseline to follow‐up were plotted against the corresponding absolute difference in weight for participants stratified by treatment group. Data are shown for participants randomized to liraglutide (red) and placebo (blue). The solid line represents the linear association between change in weight and muscle fat/muscle volume *z*‐score.

In adjusted analysis, there was a significant association between longitudinal change in muscle fat with changes in body weight, visceral adipose tissue, and abdominal subcutaneous adipose tissue (Table 3). In contrast, there was no significant association between longitudinal change in muscle fat with either total lean tissue or total thigh muscle volume. The associations of changes in body weight with changes in muscle fat and muscle volume *z*‐score across treatment groups are shown in Figure 2. In the placebo group, there was a linear association and significant correlation between changes in muscle fat and weight (*r* = 0.29, *P*‐value = 0.03). In contrast, there was no significant correlation between muscle fat change and weight change in the liraglutide group (*r* = 0.10, *P*‐value = 0.41). The correlation between change in weight and change in muscle volume *z*‐score appeared greater in the liraglutide versus placebo group (Figure 2).

**Table 3:** Adjusted association between longitudinal measures of body composition parameters and follow up muscle fat using linear mixed effect model.

Variable	Β estimate (95% CI)	*P*‐value
Weight per 1 kg increase	0.04 (0.03, 0.05)	<0.001
Visceral adipose tissue per 1 L increase	0.40 (0.28, 0.52)	<0.001
Abdominal subcutaneous adipose tissue per 1 L increase	0.13 (0.09, 0.18)	<0.001
Total lean tissue per 1 L increase	0.03 (−0.04, 0.10)	0.43
Total thigh muscle per 1 L increase	−0.04 (−0.19, 0.11)	0.59
Fat‐tissue free muscle volume *z*‐score per 1 unit increase	−0.56 (−0.75, −0.38)	<0.001

Separate linear mixed effect models were created for each body composition parameter with the body composition parameter as the exposure and muscle fat as the outcome with the inclusion of the following covariates: age, sex, race, treatment group, pre‐/post‐period, and patient ID as a random intercept.

**Figure 3:**
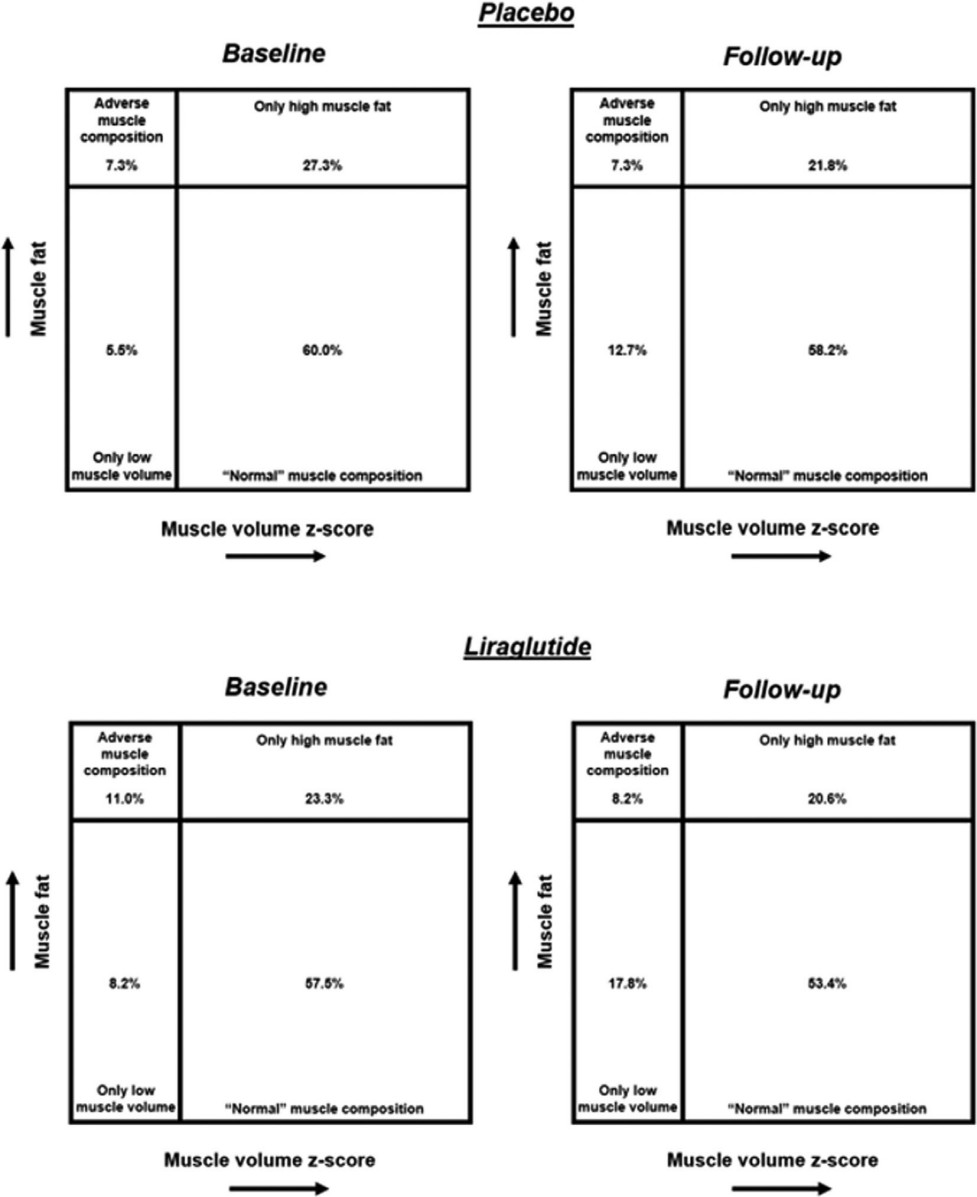
Muscle composition before and after treatment with liraglutide or placebo. Participants were analysed according to treatment group at both baseline and follow‐up. Participants were stratified based on muscle volume (low [<25th percentile of the cohort/*z*‐score <0.68] vs. high [≥25th percentile of the cohort/*z*‐score ≥0.68]) and sex‐specific muscle fat (low [women ≤8.82%, men ≤7.69%]] vs. high [women >8.82%, men >7.69%]). Adverse muscle composition was defined as low muscle volume *z*‐score and high muscle fat.

In exploratory analysis, muscle composition categories at baseline and follow‐up are shown across treatment groups (Figure 3). In the liraglutide group, the proportion of participants with adverse muscle composition decreased from 11.0% to 8.2% over follow‐up. ‘Normal’ muscle composition was found in 57.5% of participants randomized to liraglutide and this remained similar during follow‐up (53.4%). The decline in the proportion of participants with only high muscle fat (23.3% to 20.6%) was less than the increase in the only low muscle volume group (8.2% to 17.8%) observed with liraglutide. In contrast, in the placebo group, normal muscle composition and adverse muscle composition remained similar during follow‐up with a slight decrease in the proportion of participants with only high muscle fat (27.3% to 21.8%) and a concomitant increase in the proportion with only low muscle volume (5.5% to 12.7%).

## Discussion

In this pre‐specified secondary analysis of a randomized, placebo‐controlled clinical trial, we observed that liraglutide was associated with a significant reduction in muscle fat among individuals with overweight or obesity who were free of diabetes, most of whom were women. The reduction in muscle fat with liraglutide was consistent across key subgroups, including age, race/ethnicity, and BMI categories. Additionally, reduction in muscle fat was significantly correlated with reduction in overall body weight but this appeared different across treatment groups. In contrast, reduction in muscle fat was not associated with changes in lean tissue or total thigh muscle volume in adjusted analyses. Finally, liraglutide treatment, but not placebo, was associated with reduction in adverse muscle composition.

Prior studies have demonstrated that higher amounts of muscle fat are significantly associated with greater burden of traditional cardiovascular disease risk factors,^7^, ^8^ worse functional capacity,^5^, ^6^ and higher risk of adverse cardiovascular events.^9^, ^10^, ^11^ More recently, thigh muscle fat has been implicated in the development of HF, particularly HF with preserved ejection fraction. In the Health ABC study, intramuscular fat assessed by computed tomography was associated with elevated HF risk after accounting for adiposity measures including BMI, fat percentage, and visceral fat.^9^ In HF subtype analysis, muscle fat appeared to be more strongly associated with risk of HF with reduced versus preserved ejection fraction. The findings from the present study add to the existing literature by demonstrating that thigh muscle fat is modifiable with weight loss therapy such as liraglutide. The magnitude of reduction in thigh muscle fat with liraglutide (−0.24% over 40 weeks) is particularly notable as this counters the reported natural increase of muscle fat with aging of 0.4% over 5 years.^13^ Similarly, in the Lifestyle Interventions and Independence for Elders pilot (LIFE‐P) study, a physical activity intervention did not significantly change midthigh intermuscular fat over 12 months, but, when compared with the health education control, blunted the age‐related increase in intermuscular fat.^31^ The magnitude of change in muscle fat with lifestyle modification and liraglutide follows a similar pattern to that observed for weight loss with less reductions observed with diet and physical activity interventions that may be limited by adherence.^16^ The impact of liraglutide on relative changes in muscle fat (~3%) shown here, as well as its more pronounced effect on other body composition parameters evaluated previously, including liver fat (−33%), visceral adipose tissue (−11%), and weight (−5%), and their prognostic implications should be evaluated further.^21^ Prior studies have consistently demonstrated greater plasticity in liver fat content compared with other depots.^21^, ^32^ The magnitude of reduction in muscle fat was markedly less than that observed with liver fat potentially due to its less active role in systemic metabolism, less robust response to lifestyle intervention, and lower measurement variability.^21^, ^31^, ^33^, ^34^

Previous studies demonstrated that liraglutide induced weight loss through reductions in both fat mass as well as lean body mass.^35^, ^36^ However, these prior studies used dual‐energy X‐ray absorptiometry to estimate lean mass, which limits the ability to investigate specific muscles and fat infiltration of muscles.^37^ In the present study, we evaluated the effect of liraglutide on muscle fat using MRI as part of a pre‐specified secondary analysis of a clinical trial that originally examined change in visceral adipose tissue as the primary outcome. To the best of our knowledge, this is the first study to demonstrate a significant reduction in muscle fat assessed by MRI with liraglutide among adults with overweight or obesity who were free of diabetes. Whether the cardioprotective effect of liraglutide is related to improvements in body composition, like muscle fat, or other factors, such as reductions in triglycerides or increases in high‐density lipoprotein cholesterol, is not fully understood.^17^ Muscle fat may be an important target for cardiometabolic disease prevention due to its close relationship with insulin resistance, an important cardiometabolic risk factor.^38^

In parallel with the reduction in muscle fat, liraglutide also reduced muscle volume. Due to the strong association between muscle volume and body weight, a reduction in muscle volume is expected during successful weight loss, which is commonly observed with metabolic surgery.^39^ The more modest effect of liraglutide on muscle volume *z*‐score (which is normalized for sex and weight) indicates that the muscle volume lost was in line with what was expected due to the observed weight loss. The reduction in muscle fat correlated with favourable changes in other regional adiposity depots but not with changes in total lean tissue and total thigh muscle volume. These findings suggest that the favourable treatment effects of liraglutide in lowering the metabolically harmful fatty infiltration preferentially over lean tissue and skeletal muscle tissue led to a robust improvement in the proportion of participants with adverse muscle composition which decreased from 11.0% to 8.2% over follow‐up with liraglutide. This is particularly relevant considering the prognostic relevance of adverse muscle composition (high muscle fat plus low muscle volume). In an analysis of over 39 000 participants enrolled in the UK Biobank, adverse muscle composition was detected in 11% of participants and associated with all‐cause mortality even after accounting for grip strength.^14^ The prevalence of adverse muscle composition appears higher in cardiometabolic disease states such as NAFLD and is similarly associated with excess morbidity with 2‐ to 3‐fold higher risk of diabetes and coronary heart disease.^12^ The present study also provides insights into changes in muscle fat in relation to body weight changes. Disentangling these relationships has been challenging due to the association of weight loss with reductions in both adipose tissue and skeletal muscle.^36^ There was a greater decline in body weight and muscle fat with liraglutide compared with placebo but changes in these body parameters were less closely correlated with liraglutide versus placebo treatment.^21^ Taken together, the mechanisms through which liraglutide reduced muscle fat may be less related to weight loss and potentially through more direct effects but this requires further investigation. Fat depots such as muscle fat may represent a key modifiable risk factor for cardiovascular disease.

Our study findings have important clinical implications. The burden of HF is growing in the community and is driven by increasing prevalence of risk factors such as obesity.^40^, ^41^ Ongoing (ClinicalTrials.gov: NCT03574597, NCT05556512) studies are targeting obesity using weight loss therapies as a strategy to improve the overall cardiometabolic health and reduce the risk of adverse cardiovascular disease events. Obesity is a heterogeneous condition and while lean mass and different regional adiposity depots contribute to the overall body weight, the risk of HF associated with different components of the body composition are not the same.^2^, ^3^, ^4^ Specifically, regional adiposity such as visceral adipose tissue and thigh muscle fat are associated with an increased risk of HF.^9^, ^42^ Findings from the present study suggest that weight loss therapies such as liraglutide can significantly lower the amount of muscle fat and improve the overall muscle composition. Future studies are needed to examine whether improvements in muscle fat and body composition are associated with favourable subclinical cardiac phenotypes such as cardiac structure and function. Thus, findings from our study suggest that high amounts of muscle fat may be a modifiable risk factor for HF that could be targeted with effective weight loss pharmacotherapies. Future studies are needed to determine if consistent reduction in muscle fat may lower the risk of HF among overweight to obese individuals.

The strengths of the present analysis include the double‐blinded, randomized, clinical trial design and use of gold‐standard measurements of muscle composition with MRI. There are several noteworthy limitations. First, the present study included clinical trial participants, predominantly women, and findings may not be generalizable to all individuals with obesity who are not enrolled in weight loss trials. Furthermore, the present study is susceptible to selection bias due to (1) substantial attrition rate in the primary trial as is expected for studies investigating weight loss agents,^43^ and (2) this analysis included participants who completed MRI before and after treatment with liraglutide or placebo. Second, fat can be deposited in various parts of the muscle tissue, and the present study specifically assessed the average anterior thigh muscle fat infiltration measured as the T2*‐corrected proton density fat fraction of the fat‐free muscle volume (i.e., in image voxels with <50% fat within the muscle volume segmentation). This measurement targets more diffuse fat infiltration as compared with what is commonly measured using computer tomography and referred to as ‘intramuscular fat’ (by also excluding both intermuscular fat as well as larger streaks of fat inside the muscles). However, MRI cannot distinguish between intra‐ and extramyocellular lipids (IMCL and EMCL) and the effects of weight loss pharmacotherapies specifically on the amount of lipid droplets stored inside skeletal muscle cells remains unknown. Furthermore, the method used in the study has been previously evaluated and shown to have prognostic implications.^12^, ^14^ Whether the magnitude of reduction in muscle fat infiltration is clinically meaningful is unclear and requires further study. Third, the present study did not examine functional measures of muscle strength, which is a clinically important outcome. However, muscle fat and muscle volume are objective, reproducible measures that appear to have more prognostic implications, independent of the measure of muscle function, among individuals with cardiometabolic disease and those in the general population.^14^ Fourth, the effect of diet and exercise on muscle fat and comparisons with liraglutide were not performed in the present study. All participants included in the clinical trial completed a run‐in of caloric restriction and ≥150 min per week of moderate‐intensity physical activity. Fifth, adverse muscle composition is defined based on a derivation cohort from the UK Biobank imaging substudy.^14^ Overweight or obesity was part of the inclusion criteria of the present study which may lead to overestimation of adverse muscle composition. However, this bias would be expected for both treatment groups and differences are likely the result of the intervention. Finally, this study is a pre‐specified secondary analysis of a previously published trial and no adjustments were made for multiple testing.

## Conclusions

In conclusion, liraglutide administered subcutaneously once daily reduced thigh muscle fat and adverse muscle composition compared with placebo among adults with overweight or obesity who were free of diabetes and were predominantly women. Future studies are needed to confirm these observations and examine the impact of pharmacologic‐induced reductions in muscle fat on cardiometabolic disease risk.

## Funding

Clinical trial (ClinicalTrials.gov: NCT03038620) was funded by Novo Nordisk.

## Conflict of interest

Dr. Pandey is supported by the Texas Health Resources Clinical Scholarship, the Gilead Sciences Research Scholar Program, the National Institute of Aging GEMSSTAR Grant (1R03AG067960‐01), and Applied Therapeutics; has served on the advisory board for Roche Diagnostics; and has received nonfinancial support from Pfizer and Merck. Dr. Patel has served as a consultant to Novo Nordisk. Ms. Linge and Dr. Leinhard are employees and stockholders of AMRA Medical AB. Dr. Anker reports grants and personal fees from Vifor Int. and Abbott Vascular, and personal fees from Astra‐Zeneca, Bayer, Brahms, Boehringer Ingelheim, Cardiac Dimensions, Novartis, Occlutech, Servier, and Vifor Int. Dr. Butler has served as a consultant for Abbott, Adrenomed, Amgen, Array, AstraZeneca, Bayer, Boehringer Ingelheim, Bristol‐Myers Squibb, CVRx, G3 Pharmaceutical, Innolife, Janssen, LivaNova, Medtronic, Merck, Novartis, Novo Nordisk, Occlutech, Relypsa, Roche, and Vifor. Dr. Verma holds a Tier 1 Canada Research Chair in Cardiovascular Surgery; and reports receiving research grants and/or speaking honoraria from Amarin, Amgen, AstraZeneca, Bayer, Boehringer Ingelheim, Bristol‐Myers Squibb, Eli Lilly, EOCI Pharmacomm Ltd, HLS Therapeutics, Janssen, Merck, Novartis, Novo Nordisk, Pfizer, PhaseBio, Sanofi, Sun Pharma, and the Toronto Knowledge Translation Working Group. He is the President of the Canadian Medical and Surgical Knowledge Translation Research Group, a federally incorporated not‐for‐profit physician organization. Dr. Joshi has received Grant support from Novo Nordisk, as well as Amgen and Novartis. Consulting Novartis. Equity G3 Therapeutics. Dr. Neeland has received speaker and consultancy fees from Boehringer Ingelheim/Lilly Alliance, Merck, Nestle Health Sciences, and AMRA Medical; and grant support from Novo Nordisk.

## Supporting information


**Table S1.** Baseline and follow‐up characteristics stratified by treatment group.
**Table S2.** Follow‐up characteristics of participants stratified by tertiles of baseline muscle fat.
**Table S3.** Baseline characteristics of participants stratified by tertiles of percent change in muscle fat.
**Table S4.** Adjusted association of baseline characteristics with follow‐up measures of muscle fat.
**Figure S1.** Study flow diagram.
