# Supplementary material for: Effect of liraglutide on thigh muscle fat and muscle composition in adults with overweight or obesity: Results from a randomized clinical trial
Source: J Cachexia Sarcopenia Muscle. 2024 Apr 1;15(3):1072–83. doi: 10.1002/jcsm.13445 (PMC11154779; doi:10.1002/jcsm.13445)

**SUPPLEMENTAL MATERIAL**

# Supplemental Table 1. Baseline and follow-up characteristics stratified by treatment group

|  | **Liraglutide**  **(n = 73)** | **Placebo**  **(n = 55)** | **P value** |
| --- | --- | --- | --- |
| *Baseline parameters* | | | |
| Mean thigh muscle fat, % | 7.9 (6.8, 9.3) | 7.5 (6.3, 9.1) | 0.20 |
| Age, years | 49 (42, 56) | 51 (45, 56) | 0.35 |
| Female | 67 (91.8) | 51 (92.7) | >0.99 |
| Race (%) |  |  | 0.84 |
| White | 43 (58.9) | 35 (63.6) |  |
| Black | 28 (38.4) | 19 (34.5) |  |
| Systolic blood pressure, mm Hg | 131 (120, 139) | 126 (116, 135) | 0.14 |
| Diastolic blood pressure, mm Hg | 82 (75, 86) | 77 (72, 84) | 0.07 |
| Weight, kg | 100.5 (88.1, 114.9) | 101.9 (89.9, 117.2) | 0.68 |
| BMI, kg/m^2^ | 37.2 (32.3, 40.3) | 36.4 (33.8, 42.5) | 0.49 |
| Height, m | 1.6 (1.6, 1.7) | 1.6 (1.6, 1.7) | 0.63 |
| Medical history |  |  |  |
| Hyperlipidemia | 33 (45.2) | 29 (52.7) | 0.51 |
| Hypertension | 20 (27.4) | 13 (23.6) | 0.78 |
| Laboratory values |  |  |  |
| Fasting plasma glucose, mg/dL | 100 (94, 106) | 97 (90, 106) | 0.33 |
| HDL-C, mg/dL | 58 (52, 64) | 56 (48, 59) | 0.15 |
| Triglycerides, mg/dL | 99 (73, 136) | 106 (81, 142) | 0.29 |
| Body composition parameter |  |  |  |
| Visceral adipose tissue, L | 4.3 (3.0, 5.6) | 4.1 (3.4, 5.4) | 0.88 |
| Abdominal subcutaneous adipose tissue, L | 15.6 (12.0, 19.1) | 15.2 (12.8, 18.8) | 0.54 |
| Thigh muscle, L | 9.6 (8.6, 10.9) | 9.7 (8.6, 11.0) | 0.66 |
| Total lean tissue, L | 20.7 (19.1, 23.5) | 21.5 (18.7, 23.6) | 0.95 |
| Thigh muscle volume z-score | 0.3 (-0.7, 1.0) | 0.3 (-0.5, 0.9) | 0.69 |
| *Follow-up parameters* | | | |
| Mean thigh muscle fat, % | 7.6 (6.7, 8.9) | 7.5 (6.5, 9.0) | 0.82 |
| Weight, kg | 94.1 (82.2, 104.1) | 98.4 (86.3, 118.8) | 0.05 |
| BMI, kg/m^2^ | 34.6 (30.7, 37.5) | 36.5 (33.3, 41.9) | 0.01 |
| Laboratory values |  |  |  |
| Fasting plasma glucose, mg/dL | 93 (88, 99) | 98 (93, 103) | 0.01 |
| HDL-C, mg/dL | 58 (51, 67) | 54 (48, 61) | 0.06 |
| Triglycerides, mg/dL | 88 (72, 121) | 100 (75, 135) | 0.13 |
| Body composition parameter |  |  |  |
| Visceral adipose tissue, L | 3.8 (2.6, 5.1) | 4.3 (3.4, 5.0) | 0.11 |
| Abdominal subcutaneous adipose tissue, L | 14.0 (11.1, 17.8) | 15.6 (13.1, 19.2) | 0.02 |
| Thigh muscle, L | 9.2 (8.5, 10.7) | 9.6 (8.5, 11.0) | 0.31 |
| Total lean tissue, L | 20.4 (18.3, 22.5) | 21.4 (18.7, 23.0) | 0.43 |
| Thigh muscle volume z-score | 0.1 (-0.8, 0.9) | 0.3 (-0.5, 1.0) | 0.63 |
| Categorical variables are described as number (%) and compared using chi-square test. Continuous variables are described as median (25-75%) and compared using Kruskal-Wallis test.  Abbreviations: BMI, body mass index; HDL-C, high-density lipoprotein cholesterol.  *Baseline characteristics of participants stratified by treatment group were published previously in the report of the primary trial findings (Neeland, et al. Lancet Diabetes Endocrinol 2021). | | | |

# Supplemental Table 2. Follow-up characteristics of participants stratified by tertiles of baseline muscle fat

|  | **Tertile 1**  **(n = 43)** | **Tertile 2**  **(n = 43)** | **Tertile 3**  **(n = 42)** | **P value** |
| --- | --- | --- | --- | --- |
| Mean thigh muscle fat, % | 6.1 (5.4, 6.7) | 7.6 (7.2, 8.3) | 9.7 (9.1, 10.6) | <0.001 |
| Weight, kg | 89.2 (82.4, 102.7) | 96.4 (84.2, 110.5) | 102.3 (91.8, 118.0) | 0.007 |
| BMI, kg/m^2^ | 33.7 (30.4, 36.0) | 36.2 (32.4, 39.2) | 37.1 (34.5, 43.4) | 0.001 |
| Laboratory values |  |  |  |  |
| Fasting plasma glucose, mg/dL | 95 (89, 101) | 97 (90, 103) | 95 (89, 102) | 0.56 |
| HDL-C, mg/dL | 56 (51, 65) | 55 (47, 62) | 56 (50, 63) | 0.64 |
| Triglycerides, mg/dL | 86 (69, 126) | 95 (79, 132) | 98 (72, 122) | 0.31 |
| Body composition parameter |  |  |  |  |
| Visceral adipose tissue, L | 3.5 (2.6, 4.5) | 4.4 (2.9, 5.7) | 4.1 (3.4, 5.2) | 0.03 |
| Abdominal subcutaneous adipose tissue, L | 13.7 (10.5, 15.5) | 14.0 (11.4, 17.7) | 17.5 (13.8, 19.5) | 0.003 |
| Thigh muscle, kg | 9.8 (8.8, 11.1) | 9.2 (8.4, 10.8) | 9.1 (8.2, 10.3) | 0.17 |
| Total lean tissue, L | 21.5 (18.8, 22.9) | 20.4 (18.2, 22.8) | 20.7 (18.4, 22.7) | 0.76 |
| Thigh muscle volume z-score | 0.8 (0, 1.6) | -0.2 (-1.0, 0.7) | -0.2 (-1.2, 0.6) | <0.001 |
| Categorical variables are described as number (%) and compared using chi-square test. Continuous variables are described as median (25-75%) and compared using Kruskal-Wallis test.  Abbreviations: BMI, body mass index; HDL-C, high-density lipoprotein cholesterol.  *Baseline characteristics of participants stratified by treatment group were published previously in the report of the primary trial findings (Neeland, et al. Lancet Diabetes Endocrinol 2021). | | | | |

# Supplemental Table 3. Baseline characteristics of participants stratified by tertiles of percent change in muscle fat

|  | **Tertile 1**  **(n = 43)** | **Tertile 2**  **(n = 43)** | **Tertile 3**  **(n = 42)** | **P value** |
| --- | --- | --- | --- | --- |
| Mean (total range) percent change in thigh muscle fat infiltration, % | -8.2 (4.2) | -1.5 (1.2) | 4.7 (3.6) | <0.001 |
| Median (IQR) percent change in thigh muscle fat infiltration, % | -6.7 (-10.0, -5.1) | -1.5 (-2.5, -0.7) | 4.0 (1.6, 6.5) | <0.001 |
| Age, years | 48 (43, 54) | 53 (45, 57) | 50 (41, 56) | 0.40 |
| Female | 37 (86.0) | 40 (93.0) | 41 (97.6) | 0.13 |
| Race (%) |  |  |  | 0.44 |
| White | 28 (65.1) | 27 (62.8) | 23 (54.8) |  |
| Black | 13 (30.2) | 15 (34.9) | 19 (45.2) |  |
| Other | 2 (4.7) | 1 (2.3) | 0 (0.0) |  |
| Systolic blood pressure, mm Hg | 123 (116, 136) | 132 (124, 139) | 127 (115, 135) | 0.06 |
| Diastolic blood pressure, mm Hg | 80 (74, 84) | 81 (75, 86) | 79 (72, 85) | 0.78 |
| Weight, kg | 92.4 (82.1, 108.8) | 105.5 (93.5, 120.0) | 100.6 (88.6, 112.8) | 0.01 |
| BMI, kg/m^2^ | 36.1 (30.8, 39.3) | 38.3 (34.9, 42.5) | 35.9 (34.3, 40.3) | 0.08 |
| Height, m | 1.6 (1.6, 1.7) | 1.7 (1.6, 1.7) | 1.6 (1.6, 1.7) | 0.25 |
| Medical history |  |  |  |  |
| Hyperlipidemia | 24 (55.8) | 19 (44.2) | 19 (45.2) | 0.49 |
| Hypertension | 11 (25.6) | 11 (25.6) | 11 (26.2) | >0.99 |
| Laboratory values |  |  |  |  |
| Fasting plasma glucose, mg/dL | 98 (94, 106) | 100 (93, 106) | 97 (90, 106) | 0.68 |
| HDL-C, mg/dL | 54 (46, 58) | 57 (50, 61) | 59 (52, 69) | 0.18 |
| Triglycerides, mg/dL | 96 (75, 136) | 110 (82, 136) | 105 (75, 146) | 0.75 |
| Body composition parameter |  |  |  |  |
| Visceral adipose tissue, L | 4.1 (2.8, 5.3) | 4.8 (3.7, 5.6) | 4.1 (3.2, 5.0) | 0.15 |
| Abdominal subcutaneous adipose tissue, L | 14.5 (11.1, 17.4) | 17.4 (14.1, 20.5) | 14.9 (12.6, 17.9) | 0.02 |
| Thigh muscle, L | 9.5 (8.6, 10.6) | 9.7 (8.6, 11.2) | 9.7 (8.7, 10.9) | 0.89 |
| Total lean tissue, L | 21.0 (19.0, 23.2) | 21.8 (19.5, 24.0) | 20.7 (18.6, 23.5) | 0.51 |
| Thigh muscle volume z-score | 0.3 (-0.6, 1.2) | 0.3 (-0.9, 0.8) | 0.3 (-0.4, 1.1) | 0.30 |
| Categorical variables are described as number (%) and compared using chi-square test. Continuous variables are described as median (25-75%) and compared using Kruskal-Wallis test.  Abbreviations: BMI, body mass index; HDL-C, high-density lipoprotein cholesterol; IQR, interquartile range. | | | | |

# Supplemental Table 4. Adjusted association of baseline characteristics with follow-up measures of muscle fat

| **Variable** | **β estimate**  **(95% CI)** | **P value** |
| --- | --- | --- |
| Age, per 1 year increase | 0.01 (-0.01, 0.02) | 0.36 |
| Male sex (versus female) | -0.24 (-0.75, 0.27) | 0.35 |
| White (versus non-White) | -0.02 (-0.24, 0.19) | 0.83 |
| Body mass index per 1 kg/m2 increase | 0.03 (0.01, 0.05) | 0.008 |
| Systolic blood pressure per 1 mm Hg increase | 0.00 (-0.01, 0.00) | 0.40 |
| Fasting plasma glucose per 1 mg/dL increase | 0.00 (-0.01, 0.01) | 0.53 |
| HDL-C per 1 mg/dL increase | 0.01 (0, 0.02) | 0.14 |
| Baseline muscle fat per 1% increase | 0.93 (0.86, 1.00) | <0.001 |
| Standardized estimate represents the change in follow-up muscle fat per unit change in the exposure while keeping the other covariates fixed. Each of the variables listed above were included in the model. | | |

# Supplemental Figure 1. Study flow diagram.


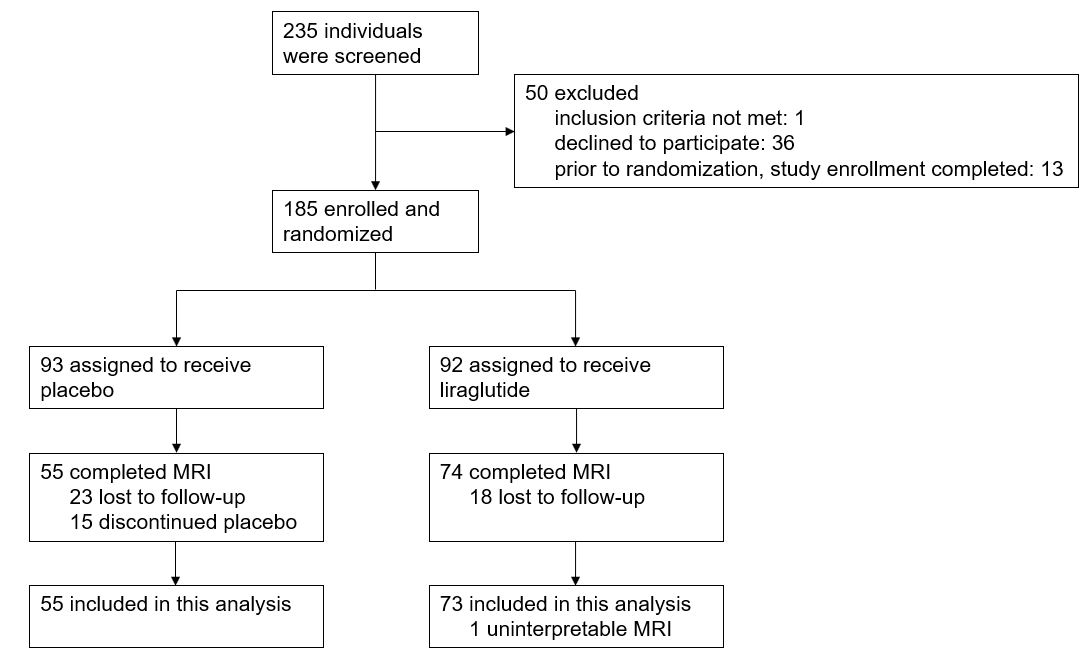

Supplement: Supplementary file 1 — Table S1. Baseline and follow‐up characteristics stratified by treatment group. Table S2. Follow‐up characteristics of participants stratified by tertiles of baseline muscle fat. Table S3. Baseline characteristics of participants stratified by tertiles of percent change in muscle fat. Table S4. Adjusted association of baseline characteristics with follow‐up measures of muscle fat. Figure S1. Study flow diagram. [file JCSM-15-1072-s001.docx]
